# Supplementary material for: Remote early detection of SARS-CoV-2 infections using a wearable-based algorithm: Results from the COVID-RED study, a prospective randomised single-blinded crossover trial
Source: PLoS One. 2025 Jun 5;20(6):e0325116. doi: 10.1371/journal.pone.0325116 (PMC12140236; doi:10.1371/journal.pone.0325116)
Supplement: S1 File — (DOCX) [file pone.0325116.s001.docx]

## S1 Appendix

Supplementary tables and figures to: *“Investigation of the effect of a wearable-based AI algorithm on the remote early detection of SARS-CoV-2 infections: results from the COVID-RED study, a prospective randomized single-blinded crossover trial”*.

**S1 Table**. Overview of Adverse Device Effects in the Safety Analysis set

|  | **Number** | **Percentage** |
| --- | --- | --- |
| **Number of subjects** | 14968 | 100 |
| **Subjects reporting any ADE** | 503 | 3.36 |
| **Subjects reporting severe ADE** | 4 | 0.03 |
| **Number of reported ADEs** | 511 | 100 |
| **ADEs considered mild** | 475 | 92.95 |
| **ADEs considered moderate** | 30 | 5.87 |
| **ADEs considered severe** | 4 | 0.01 |

ADE = Adverse Device Effect

### **Supplementary tables and figures for the 60% compliance EA and PC sets**

**S2 Table**. **Comprehensive baseline characteristics of the 60% compliance EA and PC sets**

|  |  | **EA set** | | **PC set** | |
| --- | --- | --- | --- | --- | --- |
|  |  | **Sequence 1**  **N = 1,891** | **Sequence 2**  **N = 1,920** | **Sequence 1**  **N = 2,286** | **Sequence 2**  **N= 2,333** |
| **Age (years)** | Mean (SD) | 51.3 (12.9) | 51.7 (12.6) | 50.9 (13.1) | 51.2 (12.9) |
|  | Median (IQR) | 52.0 (43.0, 61.0) | 53.0 (44.0, 61.0) | 52.0 (42.2, 61.0) | 52.0 (43.0, 60.0) |
| **Sex** | Male | 517 (27.3%) | 519 (27.0%) | 627 (27.4%) | 639 (27.4%) |
|  | Female | 1,374 (72.7%) | 1,401 (73.0%) | 1,659 (72.6%) | 1,649 (72.6%) |
| **Risk group** | High risk | 785 (41.5%) | 784 (40.8%) | 946 (41.4%) | 948 (40.6%) |
|  | Normal risk | 1,106 (58.5%) | 1,136 (59.2%) | 1,340 (58.6%) | 1,385 (59.4%) |
| **Body mass index (BMI)** | Mean (SD) | 26.6 (4.9) | 26.6 (4.9) | 26.7 (4.9) | 26.7 (5.0) |
| **Medical history** | Any risk factor | 603 (31.9%) | 609 (31.7%) | 728 (31.8%) | 713 (30.6%) |
|  | Hay fever | 196 (10.4%) | 175 (9.1%) | 224 (9.8%) | 205 (8.8%) |
|  | Moderate to severe forms of asthma | 86 (4.5%) | 85 (4.4%) | 99 (4.3%) | 103 (4.4%) |
|  | Chronic smoker’s cough | 3 (0.2%) | 7 (0.4%) | 6 (0.3%) | 7 (0.3%) |
|  | Chronic lung disease | 39 (2.1%) | 48 (2.5%) | 47 (2.1%) | 56 (2.4%) |
|  | Diabetes requiring medication | 69 (3.6%) | 62 (3.2%) | 88 (3.8%) | 79 (3.4%) |
|  | Hypertension requiring medication | 120 (6.3%) | 133 (6.9%) | 142 (6.2%) | 156 (6.7%) |
|  | Cardiac disease | 73 (3.9%) | 69 (3.6%) | 84 (3.7%) | 80 (3.4%) |
|  | Chronic kidney disease, requiring dialysis | 1 (0.1%) | 1 (0.1%) | 1 (0.0%) | 1 (0.0%) |
|  | Chronic liver disease | 3 (0.2%) | 4 (0.2%) | 3 (0.1%) | 4 (0.2%) |
|  | HIV infection | 5 (0.3%) | 1 (0.1%) | 6 (0.3%) | 2 (0.1%) |
|  | Weakened immune system/autoimmune disease | 61 (3.2%) | 62 (3.2%) | 76 (3.3%) | 69 (3.0%) |
|  | Hemoglobin disorder | 1 (0.1%) | 1 (0.1%) | 1 (0.0%) | 1 (0.0%) |
|  | Chemo, radio, or immunotherapy in past 6 months | 9 (0.5%) | 5 (0.3%) | 11 (0.5%) | 6 (0.3%) |
|  | Use of medications that weaken immune system | 25 (1.3%) | 21 (1.1%) | 31 (1.4%) | 25 (1.1%) |
|  | Had bone marrow or organ transplant in the past | 2 (0.1%) | 3 (0.2%) | 3 (0.1%) | 3 (0.1%) |
|  | Other | 191 (10.1%) | 208 (10.8%) | 238 (10.4%) | 240 (10.3%) |
| **Medication use** | Any medication use | 805 (42.6%) | 808 (42.1%) | 961 (42.0%) | 958 (41.1%) |
|  | Medication for high blood pressure/heart problems | 307 (16.2%) | 314 (16.4%) | 366 (16.0%) | 374 (16.0%) |
|  | Medication for diabetes | 67 (3.5%) | 66 (3.4%) | 86 (3.8%) | 86 (3.7%) |
|  | Medication for HIV or other chronic infectious disease | 1 (0.1%) | 6 (0.3%) | 2 (0.1%) | 7 (0.3%) |
|  | Chemotherapy, immunotherapy, and/or radiotherapy for treatment of cancer | 56 (3.0%) | 47 (2.4%) | 68 (3.0%) | 58 (2.5%) |
|  | Other medication that suppress or modulate the immune system | 523 (27.7%) | 535 (27.9%) | 619 (27.1%) | 622 (26.7%) |

EA = efficacy analysis; PC = partial compliance

**S1 Fig. Kaplan-Meier curves for the time-to-infection analysis in the EA analysis set, stratified by risk group. The strata “device-no_device” corresponds to the control condition and the strata “ no_device-device” to the experimental condition.** Survival probability refers to the probability that no event occurred.


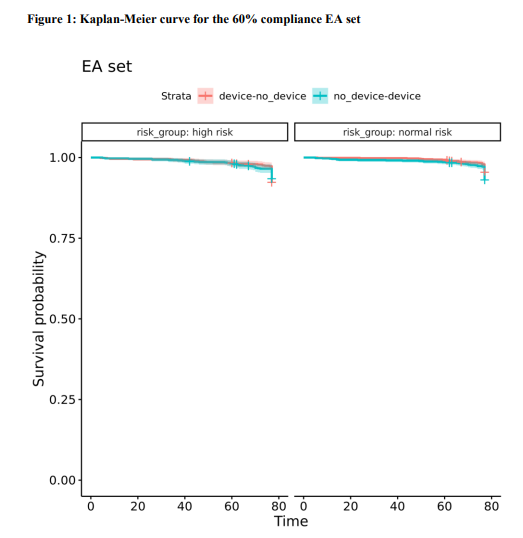


**S2 Fig. Kaplan-Meier curves for the time-to-infection analysis in the PC analysis set, stratified by risk group. The strata “device-no_device” corresponds to the control condition and the strata “ no_device-device” to the experimental condition.** Survival probability refers to the probability that no event occurred.


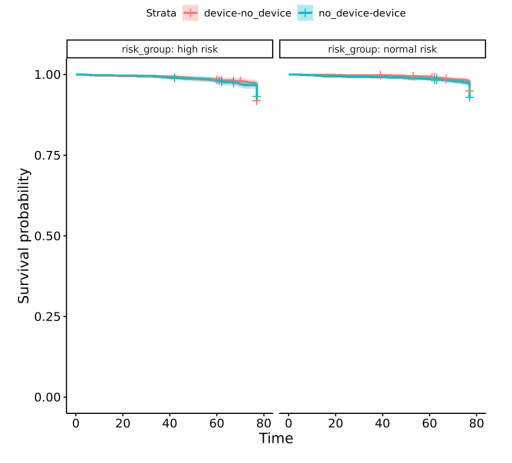


**S3 Fig.** **Kaplan-Meier curves and corresponding 95% confidence intervals for the time-to-indication analysis in the EA analysis set, stratified by risk group.** Survival probability refers to the probability that no event occurred.


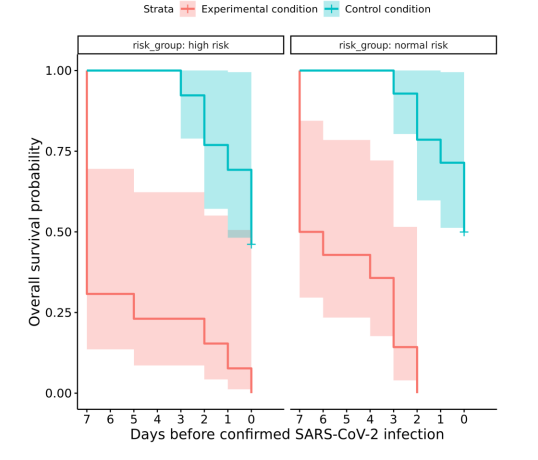


**S4 Fig.** **Kaplan-Meier curves and corresponding 95% confidence intervals for the time-to-indication analysis in the PC analysis set, stratified by risk group.** Survival probability refers to the probability that no event occurred.

**
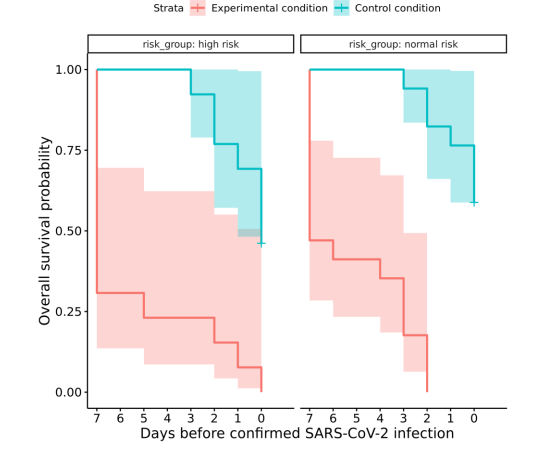
**

**S3 Table. NPV and PPV for the ever-infected analysis**

|  | **EA set** | | **PC set** | |
| --- | --- | --- | --- | --- |
|  | **Experimental** | **Control** | **Experimental** | **Control** |
| **NPV** | 93.3% | 95.2% | 90.2% | 94.6% |
| **PPV** | 6.7% | 7.6% | 6.8% | 7.9% |

EA = efficacy analysis; PC = partial compliance

### **Supplementary tables and figures for the 80% compliance EA and PC sets**

**S4 Table. Comprehensive baseline characteristics of the 80% compliance EA and PC sets**

|  |  | **EA set** | | **PC set** | |
| --- | --- | --- | --- | --- | --- |
|  |  | **Control**  **N = 1,427** | **Experimental**  **N = 1,447** | **Control**  **N = 1,965** | **Control**  **N= 2,007** |
| **Age (years)** | Mean (SD) | 52.0 (12.8) | 52.2 (12.6) | 51.4 (13.0) | 51.8 (12.8) |
|  | Median (IQR) | 53.0 (44.0, 62.0) | 53.0 (45.0, 62.0) | 52.0 (43.0, 61.0) | 53.0 (44.0, 61.0) |
| **Sex** | Male | 374 (26.2%) | 393 (27.2%) | 533 (27.1%) | 551 (27.5%) |
|  | Female | 1,053 (73.8%) | 1,054 (72.8%) | 1,432 (72.9%) | 1,456 (72.6%) |
| **Risk group** | High risk | 585 (41.0%) | 608 (42.0%) | 813 (41.4%) | 831 (41.4%) |
|  | Normal risk | 842 (59.0%) | 839 (58.0%) | 1,152 (58.6%) | 1,176 (58.6%) |
| **Body mass index (BMI)** | Mean (SD) | 26.6 (4.9) | 26.6 (4.9) | 26.6 (4.9) | 26.6 (4.9) |
| **Medical history** | Any risk factor | 447 (31.3%) | 457 (31.6%) | 625 (31.8%) | 633 (31.5%) |
|  | Hay fever | 136 (9.5%) | 132 (9.1%) | 193 (9.8%) | 183 (9.1%) |
|  | Moderate to severe forms of asthma | 64 (4.5%) | 61 (4.2%) | 85 (4.3%) | 93 (4.6%) |
|  | Chronic smoker’s cough | 1 (0.1%) | 6 (0.4%) | 4 (0.2%) | 7 (0.3%) |
|  | Chronic lung disease | 32 (2.2%) | 39 (2.7%) | 40 (2.0%) | 53 (2.6%) |
|  | Diabetes requiring medication | 52 (3.6%) | 51 (3.5%) | 74 (3.8%) | 71 (3.5%) |
|  | Hypertension requiring medication | 89 (6.2%) | 106 (7.3%) | 123 (6.3%) | 142 (7.1%) |
|  | Cardiac disease | 57 (4.0%) | 54 (3.7%) | 75 (3.8%) | 71 (3.5%) |
|  | Chronic kidney disease, requiring dialysis | 1 (0.1%) | 0 (0.0%) | 1 (0.1%) | 0 (0.0%) |
|  | Chronic liver disease | 0 (0.0%) | 2 (0.1%) | 3 (0.2%) | 3 (0.1%) |
|  | HIV infection | 3 (0.2%) | 1 (0.1%) | 5 (0.3%) | 1 (0.0%) |
|  | Weakened immune system/autoimmune disease | 46 (3.2%) | 49 (3.4%) | 65 (3.3%) | 63 (3.1%) |
|  | Hemoglobin disorder | 1 (0.1%) | 1 (0.1%) | 1 (0.1%) | 1 (0.0%) |
|  | Chemo, radio, or immunotherapy in past 6 months | 8 (0.6%) | 4 (0.3%) | 8 (0.4%) | 5 (0.2%) |
|  | Use of medications that weaken immune system | 15 (1.1%) | 16 (1.1%) | 26 (1.3%) | 24 (1.2%) |
|  | Had bone marrow or organ transplant in the past | 2 (0.1%) | 3 (0.2%) | 2 (0.1%) | 3 (0.1%) |
|  | Other | 143 (10.0%) | 147 (10.2%) | 203 (10.3%) | 211 (10.5%) |
| **Medication use** | Any medication use | 612 (42.9%) | 614 (42.4%) | 829 (42.2%) | 845 (42.1%) |
|  | Medication for high blood pressure/heart problems | 232 (16.3%) | 252 (17.4%) | 313 (15.9%) | 334 (16.6%) |
|  | Medication for diabetes | 50 (3.5%) | 54 (3.7%) | 71 (3.6%) | 78 (3.9%) |
|  | Medication for HIV or other chronic infectious disease | 0 (0.0%) | 6 (0.4%) | 0 (0.0%) | 7 (0.3%) |
|  | Chemotherapy, immunotherapy, and/or radiotherapy for treatment of cancer | 44 (3.1%) | 35 (2.4%) | 59 (3.0%) | 51 (2.5%) |
|  | Other medication that suppress or modulate the immune system | 402 (28.2%) | 402 (27.8%) | 541 (27.5%) | 552 (27.5%) |

EA = efficacy analysis; PC = partial compliance

**S5 Table. Results of the time-to-infection analysis**

|  | **EA set** | | **PC set** | |
| --- | --- | --- | --- | --- |
|  | **Control**  **N = 1,427** | **Experimental**  **N = 1,447** | **Control**  **N = 1,965** | **Experimental**  **N= 2,007** |
| **Number of subjects with first-time infection** | 81 (5.7%) | 101 (7.0%) | 119 (6.1%) | 141 (7.0%) |
| **Hazard ratio experimental vs. control** |  | 1.07 |  | 0.91 |
| **p-value of stratified log-rank test** |  | 0.15 |  | 0.21 |

EA = efficacy analysis; PC = partial compliance

**S5 Fig. Kaplan-Meier curves for the time-to-infection analysis in the EA analysis set, stratified by risk group. The strata “device-no_device” corresponds to the control condition and the strata “ no_device-device” to the experimental condition.** Survival probability refers to the probability that no event occurred.


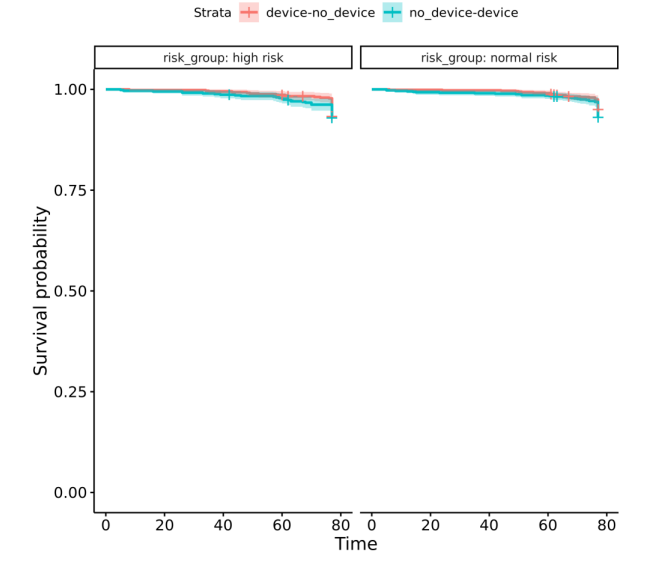


**S6 Fig. Kaplan-Meier curves for the time-to-infection analysis in the PC analysis set, stratified by risk group. The strata “device-no_device” corresponds to the control condition and the strata “ no_device-device” to the experimental condition.** Survival probability refers to the probability that no event occurred.


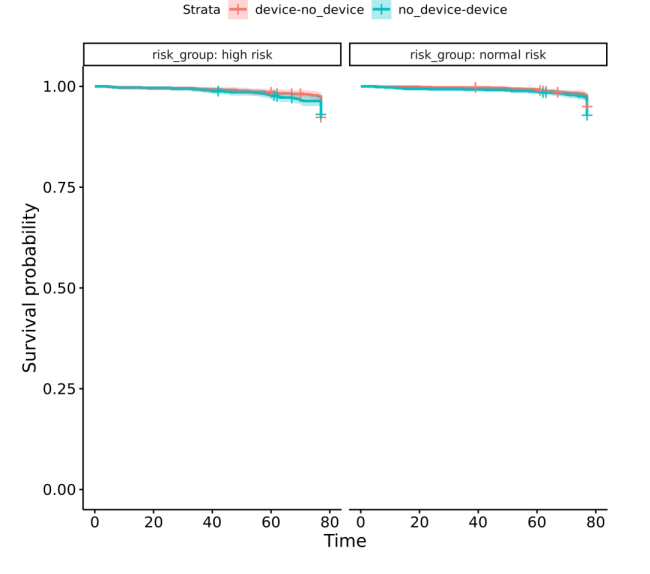


**S6 Table. Time-to-indication and corresponding p-values**

|  |  | **EA set**  **N = 23** | | | **PC set**  **N = 27** | |
| --- | --- | --- | --- | --- | --- | --- |
|  |  | **Control** | **Experimental** | **Control** | | **Experimental** |
| **Time-to-indication (days prior to positive SARS-CoV-2 test)** | Minimum | 3 | 7 | 3 | | 7 |
|  | Median | 0 | 7 | 0 | | 7 |
|  | Maximum | 0 | 0 | 0 | | 0 |
| **p-value** |  |  | < 0.001 |  | | < 0.001 |

EA = efficacy analysis; PC = partial compliance

**S7 Fig.** **Kaplan-Meier curves and corresponding 95% confidence intervals for the time-to-indication analysis in the EA analysis set, stratified by risk group.** Survival probability refers to the probability that no event occurred.


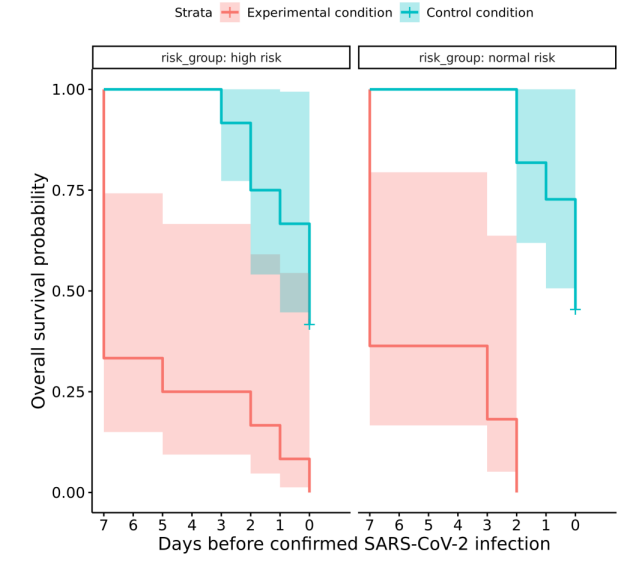


**S8 Fig.** **Kaplan-Meier curves and corresponding 95% confidence intervals for the time-to-indication analysis in the PC analysis set, stratified by risk group.** Survival probability refers to the probability that no event occurred.

**
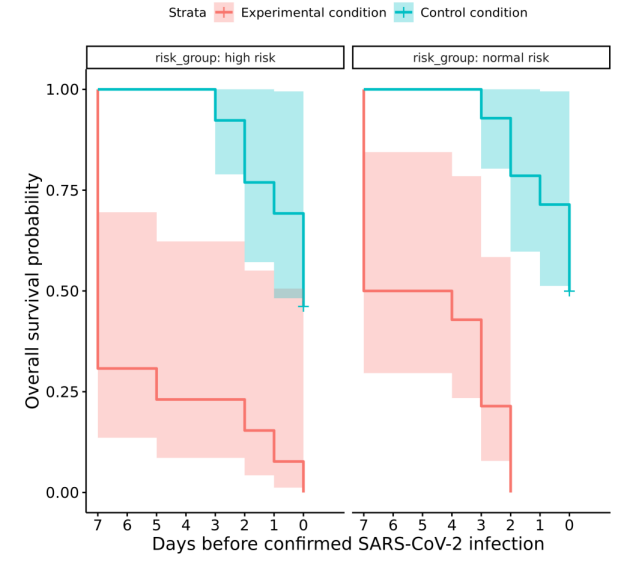
**

**S7 Table.** **Cross-tabulation of infection versus indication for the EA and PC analysis sets in the ever-infected analysis**

|  | **EA set** | | **PC set** | |
| --- | --- | --- | --- | --- |
| **Overall** | **Infected** | **Not infected** | **Infected** | **Not infected** |
| Indication positive | 138 | 1776 | 189 | 2420 |
| Indication negative | 44 | 916 | 71 | 1292 |
| **Experimental condition** |  |  |  |  |
| Indication positive | 100 | 1335 | 134 | 1801 |
| Indication negative | 1 | 11 | 7 | 65 |
| **Control condition** |  |  |  |  |
| Indication positive | 38 | 441 | 55 | 619 |
| Indication negative | 43 | 905 | 64 | 1227 |

EA = efficacy analysis; PC = partial compliance

**S8 Table. Sensitivity, Specificity, NPV and PPV for the ever-infected analysis**

|  | **EA set** | | **PC set** | |
| --- | --- | --- | --- | --- |
|  | **Experimental** | **Control** | **Experimental** | **Control** |
| **Sensitivity** | 99.0% | 46.9% | 95.0% | 46.2% |
| **Specificity** | 0.8% | 67.2% | 3.5% | 66.5% |
| **NPV** | 91.7% | 95.5% | 90.3% | 95.0% |
| **PPV** | 7.0% | 7.9% | 6.9% | 8.2% |

EA = efficacy analysis; PC = partial compliance

**S9 Table. Results of the per-day analysis when using definition 1 (diagnostic test results only)**

|  | **EA** | | **PC** | |
| --- | --- | --- | --- | --- |
|  | **Experimental** | **Control** | **Experimental** | **Control** |
| **N (number of days)** | 4 (214) | 6 (244) | 4 (214) | 7 (298) |
| **TP** | 6 | 8 | 6 | 9 |
| **TN** | 52 | 204 | 52 | 254 |
| **FP** | 149 | 17 | 149 | 17 |
| **FN** | 7 | 15 | 7 | 18 |
| **Sensitivity** | 46.2% | 34.8% | 46.2% | 33.3% |
| **Specificity** | 25.9% | 92.3% | 25.9% | 93.7% |
| **Accuracy** | 27.1% | 86.9% | 27.1% | 88.3% |

EA = efficacy analysis; PC = partial compliance

**S10 Table. Results of the per-day analysis when using definition 2 (including serology)**

|  | **EA** | | **PC** | |
| --- | --- | --- | --- | --- |
|  | **Experimental** | **Control** | **Experimental** | **Control** |
| **N (number of days)** | 11 (441) | 15 (594) | 15 (525) | 19 (742) |
| **TP** | 23 | 17 | 31 | 21 |
| **TN** | 152 | 517 | 188 | 649 |
| **FP** | 248 | 18 | 280 | 18 |
| **FN** | 18 | 42 | 26 | 54 |
| **Sensitivity** | 56.1% | 28.8% | 54.4% | 28.0% |
| **Specificity** | 38.0% | 96.6% | 40.2% | 97.3% |
| **Accuracy** | 39.7% | 89.9% | 41.7% | 90.3% |

EA = efficacy analysis; PC = partial compliance
