# Supplementary material for: Remote early detection of SARS-CoV-2 infections using a wearable-based algorithm: Results from the COVID-RED study, a prospective randomised single-blinded crossover trial
Source: PLoS One. 2025 Jun 5;20(6):e0325116. doi: 10.1371/journal.pone.0325116 (PMC12140236; doi:10.1371/journal.pone.0325116)
Supplement: Attachment — Submitted filename: Response-to-reviewers.docx [file pone.0325116.s005.docx]

**Response to reviewer comments on manuscript “A wearable-based AI algorithm for the remote early detection of SARS-CoV-2 infections: results from the COVID-RED study, a prospective randomised single-blinded crossover trial”**

*Additional journal requirements*

- Please ensure that your manuscript meets PLOS ONE's style requirements, including those for file naming.

*Author response:*

*The revised version of the manuscript adheres to the indicated style requirements.*

- We note that the current version ethics document has masked details. Before we proceed with your submission, please upload a clean version of the ethics document.

*Author response:*

*A clean version of the ethics document has been uploaded.*

- During our internal review of your submission, we noted that your clinical trial registration number (NL9320) cannot be found on the the Netherlands Trial Register. Please provide the correct registration number and a link to the online registration page if possible. Thank you for your attention this request.

*Author response:*

*We realized that the website of the Netherlands Trial Register has been discontinued, which indeed makes it difficult to find the registered trial online. The trial number is still correct, and can be verified through the following two webpages:* [Remote Early Detection of SARS-CoV-2 infections | Research with human participants](https://onderzoekmetmensen.nl/en/trial/23180); [ICTRP Search Portal](https://trialsearch.who.int/Trial2.aspx?TrialID=NL-OMON23180).

- Please note that funding information should not appear in the Acknowledgments section or other areas of your manuscript. We will only publish funding information present in the Funding Statement section of the online submission form. Please remove any funding-related text from the manuscript.

*Author response:*

*The funding-related text has been removed from the manuscript. Updated funding information is provided in the online submission form and in the cover letter.*

- We note that one or more of the authors are employed by a commercial company: Ava AG, Julius Clinical, Haleon, Dr Risch
  - Please provide an amended Funding Statement declaring this commercial affiliation, as well as a statement regarding the Role of Funders in your study. If the funding organization did not play a role in the study design, data collection and analysis, decision to publish, or preparation of the manuscript and only provided financial support in the form of authors' salaries and/or research materials, please review your statements relating to the author contributions, and ensure you have specifically and accurately indicated the role(s) that these authors had in your study. You can update author roles in the Author Contributions section of the online submission form.
  - Please also include the following statement within your amended Funding Statement.

“The funder provided support in the form of salaries for authors [insert relevant initials], but did not have any additional role in the study design, data collection and analysis, decision to publish, or preparation of the manuscript. The specific roles of these authors are articulated in the ‘author contributions’ section.”

*Author response:*

*The funder of this study (Innovative Medicines Initiative) did not provide support for authors in the form of salaries. Author salaries were paid by the various consortium partners that authors are affiliated with. We have therefore not fully included the above text in the amended Funding Statement. The statement does indicate that the* *funding body had no role in the design of the study; the collection, analysis, and interpretation of the data; and the writing of the manuscript. In addition, the funding statement indicates that the manuscript only reflects the personal views of the stated authors, and not those of the commercial companies they are affiliated with.*

- If your commercial affiliation did play a role in your study, please state and explain this role within your updated Funding Statement.
- Please also provide an updated Competing Interests Statement declaring this commercial affiliation along with any other relevant declarations relating to employment, consultancy, patents, products in development, or marketed products, etc.
- Within your Competing Interests Statement, please confirm that this commercial affiliation does not alter your adherence to all PLOS ONE policies on sharing data and materials by including the following statement: ""This does not alter our adherence to PLOS ONE policies on sharing data and materials.” (as detailed online in our guide for authors http://journals.plos.org/plosone/s/competing-interests).
- If this adherence statement is not accurate and there are restrictions on sharing of data and/or materials, please state these. Please note that we cannot proceed with consideration of your article until this information has been declared.
- Please include both an updated Funding Statement and Competing Interests Statement in your cover letter. We will change the online submission form on your behalf.

*Author response:*

*The Funding Statement and Competing Interest Statements have been updated following the editor’s instructions. Updated versions are in the cover letter.*

- One of the noted authors is a group or consortium: COVID-RED consortium
  - In addition to naming the author group, please list the individual authors and affiliations within this group in the acknowledgments section of your manuscript. Please also indicate clearly a lead author for this group along with a contact email address.

*Author response:*

*Members of the COVID-RED consortium are now listed in the acknowledgements section of the manuscript. The lead author of this group is indicated in line 521.*

- Please include a caption for figure 1.

*Author response:*

*A caption is included in the revised manuscript. The figure number was adjusted, and this is now figure 3. Other figure numbers have been adjusted accordingly.*

- Please upload a new copy of Figure 2 as the detail is not clear.

*Author response:*

*A new copy is uploaded with the revised manuscript.*

- Please include captions for your Supporting Information files at the end of your manuscript, and update any in-text citations to match accordingly.

*Author response:*

*Captions and updated in-text citations were included in the revised manuscript.*

- We note that the original protocol file you uploaded contains a confidentiality notice indicating that the protocol may not be shared publicly or be published. Please note, however, that the PLOS Editorial Policy requires that the original protocol be published alongside your manuscript in the event of acceptance. Please note that should your paper be accepted, all content including the protocol will be published under the Creative Commons Attribution (CC BY) 4.0 license, which means that it will be freely available online, and any third party is permitted to access, download, copy, distribute, and use these materials in any way, even commercially, with proper attribution.
  - Therefore, we ask that you please seek permission from the study sponsor or body imposing the restriction on sharing this document to publish this protocol under CC BY 4.0 if your work is accepted. We kindly ask that you upload a formal statement signed by an institutional representative clarifying whether you will be able to comply with this policy. Additionally, please upload a clean copy of the protocol with the confidentiality notice (and any copyrighted institutional logos or signatures) removed.

*Author response:*

*The study protocol is public domain in compliance with EU regulations and has been previously published, as indicated in line 145 of the revised manuscript. The currently uploaded version of the protocol file does not contain a confidentiality notice and can be published alongside the manuscript in the event of acceptance.*

*Additional Editor comments*

The submitted manuscript reports on a rigorous and extensive work. My major concern is the inconsistency between the results and the conclusions. The findings will be much more useful for future research if they are commented faithfully, without claims that are not supported by the data. Please ensure that this is revised throughout the whole manuscript.

*Author response:*

*We thank the editor for their compliments on the work performed. Textual adjustments have been made throughout the revised manuscript to avoid any claims that are not supported by the data.*

*Reviewer 1*

The trial design is quite interesting, as it involves a crossover with two groups over two periods. The statistical analysis is well-defined and rigorously applied, and the results are well-reported. However, the specificity of the experimental protocol was notably low and disappointing. This has important implications, as a protocol that very frequently returns positive results tends to overestimate infections. While it is preferable to report an infection rather than miss it, the extremely low specificity raises concerns. I am resistant to say that a device would necessarily be effective or (cost effective) in these circumstances. The manuscript does point to the low specificity and says that future finetuning mechanisms are needed. I would like to see more discussion on how to do finetuning and to evaluate the effectiveness.

An initial suggestion would be to use the Youden index to establish an appropriate cutoff, ensuring the protocol is more effective in practice, although finetuning most likely involves more effort than simply using this index.

*Author response:*

*We thank the reviewer for their kind words about the manuscript. We agree that the extremely low specificity of the algorithm is a limitation to the study. However, the current study’s scope was to report on the performance of the algorithm that was applied in real-time during the trial, which resulted in a high number of false positives. Further research, including also the forthcoming paper mentioned in Line 427 of the manuscript, will focus on methods for improving the algorithm. We have also shared as much data from this study as possible so that others can learn from our experiences.*

*We have added some further discussion (e.g., line 430) on how to finetune the algorithm in the future. Cost-effectiveness was discussed in another (preprint) publication, which has now been referenced in the revised discussion (line 420).*

**Abstract**

- The phrase "Performance evaluated using measures of diagnostic accuracy" is vague. It would be helpful to provide more specific details on the evaluation metrics used.
- The reported specificity of "0.8–4.4%" seems likely to be a typo. I believe it should be "0.8%–4.2%." Please verify this.
- The term "algorithm ingesting data" is somewhat technical (my impression) for an abstract. To reach a broader audience, I suggest rephrasing it to "data was used as input to the algorithm" or something along these lines.

*Author response:*

*Slight adjustments to the abstract were made to improve clarity and fix the typo.*

**Introduction**

- "Relatively young sample of individuals" should be revised to "sample of relatively young individuals."
- Line 194: Correct the phrase to "daily diary."

*Author response:*

*Both these textual adjustments have been made in the revised version of the manuscript.*

**Methodology**

- Was a sample size analysis performed? While the number of people enrolled seems sufficiently large, there is a question about whether the study period was sufficient to observe a number of outcomes. I recommend including information on sample size analysis conducted.

*Author response:*

*Section 4.4 of the appended protocol provides information on the sample size calculations. Traditional power calculations could not be performed due to the statistical methodology, and the fact that power was highly dependent on volatile factors (including the infection rate, which was unknown and constantly changing with new variants of the virus). We have therefore decided not to report on the sample size in the current manuscript.*

- The description of "per-day analysis" is unclear. I understand it as calculating the number of infected days across all participants, which serves as a measure of person-time. Please verify if this interpretation is correct and consider clarifying the description in the text and in Table 5.

*Author response:*

*This interpretation is correct. We have adjusted the text and added rows to Table 5 for additional clarifications.*

- Another suggestion is to report the Youden index (sensitivity + specificity - 1) and indicate under which circumstances this index was positive (or exceeded a defined cutoff).

*Author response:*

*We agree with the reviewer that methods such as the Youden index could have been used for improving the study algorithm and reporting on its comparative performance. However, no cutoff was defined during the study itself, and we currently are not able to further investigate algorithm performance with various cutoffs as the algorithm’s underlying code and probability outputs are proprietary. We have therefore chosen not to report the Youden index in the current paper, but have instead listed the use of this index as a suggestion for algorithm improvement in line 431.*

**Results**

- Figure 2 requires higher image resolution or better text contrast. When I attempted to download it to try to see in better resolution, only the Figure 3 was downloaded. I double checked it but I am not sure why this happens.

*Author response:*

*A new version of the figure has been uploaded with the revised manuscript.*

- In S1 Appendix, I suggest adding to the captions that "survival probability" refers to the probability that the event has not occurred.

*Author response:*

*We thank the reviewer for this suggestion and have added figure legends in the appendix accordingly.*

**Discussion**

As indicated above, I would like to see a clearer outline of how to fine-tune the protocol, as well as metrics for a cost-effectiveness analysis.

*Author response:*

*The discussion has been extended with an additional outline of methods for improving the algorithm (e.g., line 429). Cost-effectiveness analysis is beyond the scope of the current paper, but is mentioned in Line 420, with a reference to a (preprint) publication on the economic evaluation of the study.*

*Reviewer 2*

**Main strengths of the paper:**

1) Innovative concept and relevance

The COVID-RED study embodies an innovative approach by leveraging wearable technology and artificial intelligence (AI) to address a critical public health issue: the early detection of SARS-CoV-2 infections. This research is particularly relevant in light of the COVID-19 pandemic, where the ability to identify infections during the pre-symptomatic or asymptomatic phase could significantly curb transmission rates. The study bridges advanced AI algorithms with wearable medical devices, showcasing a futuristic and interdisciplinary method for monitoring public health. This integration of technology with healthcare represents a paradigm shift from traditional diagnostics to more proactive and predictive health monitoring.

2) Robust study design

The study design is meticulously structured to ensure reliability and scientific rigor:

- Randomized Controlled Trial (RCT): A prospective, single-blinded, two-period, two-sequence, randomized crossover trial was employed. This robust design minimizes biases and allows a direct comparison between the experimental (wearable-based AI algorithm) and control (symptom-based) conditions.
- Large Sample Size: The inclusion of 17,825 participants is a remarkable achievement, providing high statistical power and the ability to generalize findings across populations.
- Crossover Nature: The two-period crossover design ensures that each participant serves as their control, reducing variability and enhancing the internal validity of the results.
- These features collectively underscore the study's scientific robustness and provide a solid foundation for its conclusions.

3) Use of cutting-edge wearable technology

The article highlights the use of the Ava bracelet, an FDA-cleared and CE-certified wearable device originally designed for fertility tracking, repurposed for infection detection. The bracelet's ability to measure multiple physiological parameters, including respiratory rate, heart rate, heart rate variability, wrist-skin temperature, and skin perfusion, is a testament to its advanced capabilities. The study emphasizes the bracelet’s potential for continuous health monitoring, enabling real-time alerts for possible infections. Repurposing existing technology for public health surveillance is a forward-thinking approach that maximizes the utility of available resources. The wearable is user-friendly, requiring participants to wear it during sleep and synchronize data via a mobile app, ensuring convenience and accessibility.

4) Significant findings supporting early detection

The article demonstrates that the experimental wearable-based AI algorithm outperforms symptom-only models in detecting SARS-CoV-2 infections. The algorithm achieved high sensitivity (93.8-99.2%) in detecting infections during the study period, ensuring that most infected individuals were identified. Alerts based on the wearable device were issued significantly earlier (median of 7 days before a positive test) compared to symptom-based alerts, which had no prior warning. These findings underscore the potential of wearable devices to serve as an early warning system, particularly valuable during infectious disease outbreaks.

5) Comprehensive Performance Evaluation

The article provides a detailed evaluation of the experimental algorithm's performance through various metrics:

- Sensitivity vs. Specificity: While acknowledging the trade-offs (high sensitivity but low specificity), the study emphasizes the algorithm’s strengths in detecting infections, particularly in pre-symptomatic phases.
- Multi-Faceted Analysis: Different analytical approaches—time-to-infection, time-to-indication, and per-day analyses—offer a comprehensive assessment of the algorithm’s utility.

6) Real-World Applicability

The study's context—conducted during the COVID-19 pandemic—adds real-world relevance to its findings. Unlike retrospective studies, this trial evaluated the algorithm’s effectiveness in real-time, simulating actual scenarios where timely detection is critical. By combining physiological data with laboratory-confirmed SARS-CoV-2 infections, the study achieves a holistic approach to infection detection.

*Author response:*

*We thank the reviewer for their kind feedback on the strengths of the manuscript.*

**Areas of improvement**

1) Specific challenges

One of the study's key findings is the low specificity of the experimental algorithm (0.8–4.4%) compared to the symptom-only control algorithm (65–66.4%). While high sensitivity ensures most infections are detected, the high rate of false positives has significant implications. Frequent false alerts can overwhelm testing resources and create undue anxiety among users, reducing trust in the system. Excessive testing for false positives may strain public health systems, particularly during pandemics.

Suggestions:

- Incorporate additional parameters, such as behavioral data or exposure history, to refine the algorithm.
- Use advanced machine learning techniques, such as ensemble methods or Bayesian inference, to balance sensitivity and specificity better.
- Develop dynamic thresholds that adapt to local infection prevalence, reducing false positives in low-risk settings.

*Author response:*

*We thank the reviewer for their suggestions for improving the algorithm and agree that these additional strategies could be used for improving algorithm specificity. The scope of the current paper was to report on the performance of the algorithm that was developed during the trial with a large proportion of the data collected and subsequently applied in real-time during in the latter part of the trial. While this allowed for a valuable prospective evaluation of the real-world performance of the algorithms in the same population, the algorithm can be further improved and evaluated in a retrospective manner using all the collected data and through methodological improvements such as those suggested by the reviewer. Given the scope of this paper, these suggestions were not applied. The reviewer’s suggestions have been taken into account when revising the discussion of the manuscript, which now includes references to some of the suggested methods.*

2) Algorithm adaptability

The study design required freezing the algorithm at the start of each study period, which limited its ability to adapt to changing epidemiological conditions or incorporate newly available data. The fixed algorithm could have reduced the model's effectiveness, especially as new variants of SARS-CoV-2 emerged or vaccination rates increased.

Suggestions:

- Implement continuous learning mechanisms to allow the algorithm to evolve in response to new data, such as emerging variants or shifting symptoms.
- Explore federated learning, where the algorithm learns from decentralized data sources without compromising user privacy, to increase adaptability.

*Author response:*

*The study team was aware of the limitations of the algorithm development process but deemed the approach applied in this trial as the most feasible and suitable given the aim of the study and the challenging conditions and timeframe within which the trial was conducted. As discussed in line 436 of the manuscript, continuous learning mechanisms have been considered by the study team as a method for improving the algorithm in the future. However, the application of such methods is beyond the scope of the current paper. Federated learning and continuous learning mechanisms were not deemed feasible or suitable for the current study given logistical, technological and methodological constraints.*

3) Limited focus on specific infections

The algorithm was designed to detect SARS-CoV-2 infections but lacked the ability to differentiate between COVID-19 and other respiratory illnesses. Many false positives could result from physiological changes due to non-COVID-19 infections, such as the flu or common cold. The study did not explore the potential of wearable devices for broader infectious disease surveillance.

Suggestions:

- Expand the dataset to include physiological data from individuals with other respiratory infections.
- Train the algorithm to distinguish between SARS-CoV-2 and other illnesses, enhancing its diagnostic utility.
- Investigate the algorithm’s potential for detecting comorbid conditions or other diseases that impact similar physiological parameters.

*Author response:*

*While thoroughly considered, logistical, budgetary, and methodological constraints unfortunately meant that the study set-up and data collection did not account for investigating data from other respiratory infections, or to identify other illnesses or comorbid conditions. However, we fully agree that this likely affected the performance of the algorithm and that future studies could greatly benefit from having data on other outcomes available, as we also discuss in line 435 of the manuscript.*

4) Generalizability of findings

Although the study's sample size was large, its findings may have limited applicability to diverse populations:

- Demographic Limitations: Most participants were from the Netherlands, potentially limiting generalizability to populations with different demographics, healthcare systems, or COVID-19 prevalence rates.
- Age Range and Health Status: The study included primarily younger and healthier individuals, with fewer older or high-risk participants.

Suggestions:

- Conduct follow-up studies in diverse geographic locations and healthcare contexts to ensure broader applicability.
- Stratify results by demographic factors (e.g., age, gender, pre-existing conditions) to understand how the algorithm performs across subgroups.

*Author response:*

*While we agree that the current study’s results may not directly translate to geographical areas with vastly different demographics, healthcare systems, or COVID-19 prevalence rates, we still believe that findings can be generalizable to a broader context. Our sample was quite diverse, and we intentionally recruited participants from both “high risk” as “normal risk” populations (line 159). While beyond the scope of the current study, we agree with the reviewer that future studies could be performed in more diverse locations, and that such studies could further stratify results by demographic factors and explore those dynamics.*

*Reviewer 3*

This manuscript presents the COVID-RED study, which investigates the use of the Ava bracelet for the early detection of SARS-CoV-2 infections in real-time. The study is noteworthy for its scale, involving approximately 20,000 participants spanning diverse age groups and genders. The results demonstrate that integrating wearable data significantly enhances the sensitivity of COVID-19 detection, although it comes at the cost of reduced specificity compared to the control group without wearable data. The study is well-designed, and the dataset is particularly valuable due to its large sample size. The analyses are robust, and the manuscript is clearly written and well-structured.

However, my primary concern lies in the manuscript's alignment with its title, "A wearable-based AI algorithm for the remote early detection...". While the study focuses on the advantage of including wearable data, there is insufficient information about the AI algorithm itself. Details on the algorithm—both with and without wearable data—are largely absent. Since algorithmic performance can significantly impact predictive outcomes, it is essential to provide more information about the models used, their training processes, and any comparisons made to alternative algorithms. Without this critical component, it is challenging to assess whether the best-performing algorithms were employed for both experimental and control groups. I recommend including these details to enable a more comprehensive evaluation of the study.

*Author response:*

*We thank the reviewer for their kind words on the study design and value of the dataset. The scope of the current paper was to report on the performance of the algorithm that was developed during the trial and applied in real-time in period 2 of the trial. More information on the training process of this experimental algorithm has been provided by adding a paragraph to the methods section of the manuscript (line 234). As indicated in line 195, the control algorithm mimicked standard care, which meant that alerts were generated when symptoms were reported. Algorithm development was done by Ava and the code underlying the algorithms is considered proprietary. We are therefore not able to share more detailed information about the algorithms at this point.*

*Reviewer 4*

Thank you for your collective efforts to advance the use of wearable devices in infectious disease surveillance. Here are some points to consider regarding your paper:

- There is no caption in the text for Figure 1. Other figures also seem to be incorrectly referenced in the body of text (1 for 2, 2 for 3, 3 for 1).

*Author response:*

*Figure captions and numbering were updated in the revised version of the manuscript.*

- Line 220: A brief description of the ML algorithm used would be helpful.

*Author response:*

*We thank the reviewer for this suggestion and have added a brief description of the algorithm in the methods section (Line 234).*

- Line 226: How were the participants who did not seek testing classified?

*Author response:*

*When no testing was sought, the participant was considered to not have a positive test (i.e., they were considered negative). We were not able to collect any data that provided further information on the status of these participants.*

- Line 229: How were the participants who did not return the kits classified? (completed follow-up/drop-out/…)

*Author response:*

*When no result of the test was available, the participant was considered to not have a positive test, as further data on unreceived tests was not available. Our efforts to increase compliance and decrease drop-out throughout the study will be discussed in a forthcoming manuscript.*

- Line 232: repeated “the end of”

*Author response:*

*A textual adjustment has been made.*

- Line 309: How was the beginning of infection determined in asymptomatic cases?

*Author response:*

*The per-day analysis did not consider asymptomatic cases per the study design. This is outlined in line 300.*

- Line 336: Table legends should be placed above the body of the table (not below).

*Author response:*

*Formatting adjustments have been done in a revised version of the manuscript.*

- Line 381: Please provide the value of AUC in addition to the stated metrics (line 316).

*Author response:*

*We thank the reviewer for this suggestion, but have ultimately decided to not present this metric in the table. The current manuscript presents a variety of metrics that all show that the experimental and control algorithm showed very different behavior, which the AUC would likely convey as well. In addition, calculation of the AUC would require for us to assess algorithm performance at various thresholds, which is not possible due to the algorithm’s underlying code and probability outputs being proprietary. Future publications on the algorithm may further investigate the performance at various cutoffs, but this is beyond the scope of the current manuscript.*

- Line 384: The minimum and median time-to-indication for the experimental condition in both groups are seven days (Line 354). I see that it is stated in line 276, “The clinical endpoint of interest was the first red alert indicator in the week prior to the date at which the SARS-CoV-2 infection was confirmed through testing”. Here, the question is raised regarding the basis upon which this timeframe (one week or seven days) is determined. If a different duration was chosen, supposedly 3 days or 14 days, would we have seen the median to be 3 days and 14 days, respectively? Considering the high false positive rate of the model, more explanation could be helpful to differentiate it from a random model, stochastically raising alarms. Such a random model could have still achieved similar results of seven days, rendering the significance of this finding obsolete (Line 384).

*Author response:*

*The timeframe of seven days, as defined in line 281, was chosen at the start of the study based on knowledge about SARS-CoV-2 infections that was available at that time, which was limited due to the quickly changing epidemiological context. Other time-frames were not considered in the current study and data availability does not allow for us to investigate what would have happened when another duration would have been chosen. However, given that the experimental algorithm had low specificity and generated much more alerts than the control condition, this definitely contributed to a much earlier indication time than in the control condition. In line 397 in the discussion, we discuss how the early detection of the experimental algorithm could be interpreted as a result of its high sensitivity and low specificity rather than its ability to accurately detect infections so early.*

- Line 390: It is acknowledged that both sensitivity and specificity improved using the second definition. Although the experimental condition achieved a sensitivity superior to that of control, specificity was yet considerably lower. So, it would be incorrect to deduce that the experimental algorithm performed better “… in comparison to the control algorithm”. The accuracy is still lower than that of the control (50.2% Vs. 90.1%). A comparison cannot be based merely on sensitivity. It would be much more helpful if the area under the curve is presented for each algorithm as it was previously planned in the methods section (line 316) to help make a comparison.

*Author response:*

*The statement in line 390 has been removed and the first paragraph of the discussion has been rewritten to better reflect the low specificity of the experimental algorithm. As discussed above, we decided to not present the AUC for further comparisons. The results presented clearly indicate that both algorithms behaved very differently, and a decision on which of the two is better would be dependent on specific goals. If the goal would be to not use many resources or tests, the control algorithm would be better, but this would mean not catching many people with the disease in the early stages (see the time-to-indication analysis). The experimental algorithm, on the other hand, would amount to a large use of resources for testing, but would make sure that almost no infections are missed, which was an important consideration in the development of the algorithm. In a future pandemic, research can focus on finding a more optimal combination of sensitivity and specificity, but this is beyond the current manuscript’s scope. Instead, we have included a discussion on finding a more optimal balance between sensitivity and specificity in lines 431-436.*

- Line 408: “This might be explained by the algorithm’s inability to differentiate between SARS-CoV-2 and other (respiratory) infections”. A much better explanation is provided on Line 422. Other infections, given that 67.7% of subjects received a red alert at least once, is a farfetched explanation.

*Author response:*

*Some wording is added to state that the explanation provided would not explain all false positives, and that other potential reasons for the algorithms’ low specificity (including the one outlined in line 439) should be considered.*

- Line 460: “… with the experimental algorithm achieving high sensitivity”. Partial representation of the findings-should be revised. Nonetheless, the study can “confirm a potential future role of wearable devices in infectious disease surveillance” even without an outstanding performance of the algorithm.

*Author response:*

*Partial representation of findings was revised by added that specificity was low, while keeping in the statement on the potential role of wearable devices in infectious disease surveillance.*
