# Supplementary material for: Remote early detection of SARS-CoV-2 infections using a wearable-based algorithm: Results from the COVID-RED study, a prospective randomised single-blinded crossover trial
Source: PLoS One. 2025 Jun 5;20(6):e0325116. doi: 10.1371/journal.pone.0325116 (PMC12140236; doi:10.1371/journal.pone.0325116)
Supplement: Attachment — Submitted filename: response to reviewers MAR25.docx [file pone.0325116.s006.docx]

**Response to reviewer comments on manuscript “A wearable-based AI algorithm for the remote early detection of SARS-CoV-2 infections: results from the COVID-RED study, a prospective randomised single-blinded crossover trial”**

*Editor Comments*

I apologise for taking a long time to make a decision. I believe the manuscript was greatly improved upon revision, however I also agree with Reviewers 1 and 4. If this work is to contribute to the development of the relative research domain, it should provide the details necessary for reproducibility.

*Author response:*

*We thank the editor for their kind words on the improvements made during the first round of revisions. We provide responses to the comments from Reviewers 1 and 4 below. We have provided as much detail as possible for reproducibility and have made anonymised study data publicly available for future researchers. By doing this, we believe this works contributes to the scientific literature in the field.*

*Reviewer 1*

Several questions and concerns were raised in my review of the first submission regarding the methodology and the evaluation of the results in assessing the algorithm. I had hoped to see these questions addressed to better understand its performance and the reasons for the observed low specificity. This understanding is really important for a proper evaluation of the results. In fact, another review noted that the title of the manuscript specifically refers to the algorithm. Unfortunately, many of these issues cannot be fully addressed due to the proprietary nature of the algorithm, as stated in the authors’ response. Furthermore, additional evaluations are not possible for the same reason. I see this lack of description regarding the algorithm as a major weakness.

*Author response:*

*The reviewer correctly notes that the proprietary nature of the algorithm poses a limitation to our study. Despite this limitation, the authors still feel that the current manuscript adds to the scientific literature by providing such a large-scale evaluation of the use of a wearable device for SARS-CoV-2 infection detection. To provide more explanation for the low specificity of the algorithm, we have added some clarification about the chosen probability threshold in the development of the algorithm in line 245. In addition, we have added a statement about this to the discussion (line 423) as well. These additions hopefully better clarify the reasons for the high sensitivity and low specificity of the algorithm better, and these insights might benefit researchers working on similar projects in the future.*

*Regarding the title of the current manuscript, we understand that this might refer to the AI algorithm itself too strongly. However, we do feel it is worthwhile to mention it in the title as the wearable-based algorithm is the main intervention that is being evaluated in this trial and thus informs the reader accordingly. A potential alternative title could be “Remote early detection of SARS-CoV-2 infections using a wearable-based algorithm: results from the COVID-RED study, a prospective randomised single-blinded crossover trial”. If the editor has a preference for this alternative title, or has an alternative suggestion, we are open to discussing this modification.*

*Reviewer 2*

During the first review, I have already accepted the original submission with specific comments and do not have more remarks to add.

*Author response:*

*We thank the reviewer for their approval of the original and current submission.*

*Reviewer 3*

All my comments have been addressed by the authors and I recommend this paper to be accepted by PLOS One journal.

*Author response:*

*We thank the reviewer for this recommendation.*

*Reviewer 4*

Dear authors,

Thank you for your responses. I appreciate your effort in addressing the raised concerns and making the necessary revisions. However, there still remains the issue regarding the reporting of the ROC AUC.

Research is built on the ability of others to understand and evaluate findings. Allowing for the reproducibility of your methodology to be disregarded due to the proprietary nature of your product, still either intentionally or unintentionally omitting standard performance metrics, especially one as widely used as AUC, compromises transparency. Even if the internal workings of an algorithm or the probability outputs are proprietary, reporting aggregate performance metrics does not reveal trade secrets.

Moreover, as you have elaborated in the introduction by reviewing other studies and comparing their reported AUCs, it is standard practice to report this aggregate metric. Future studies, as recommended in your discussion, would not have a sound basis to compare their model with the one developed here if this metric is omitted. This aggregate metric allows readers and researchers to fairly compare the proposed method to others. Not reporting this metric leaves a gap in the evaluation of the method's effectiveness. This issue directly affects the evaluation of your method's performance and the validity of the comparisons drawn thereafter. Addressing it is essential for upholding the manuscript's scientific rigor.

Finally, regarding the technical aspect of this issue, whatever environment is employed to develop an RNN can also be used to calculate AUC, as it provides “probability outputs” for the model’s predictions.

I should also remind you that the last line of methods (line 320) still lists AUC as a metric you were supposed to calculate and report.

Best regards

*Author response:*

*We thank the reviewer for their suggestions and agree that reporting the AUC would be an improvement of the manuscript. As we indicated in our initial rebuttal, this performance metric was not initially considered for reporting due to its limited value for the evaluation of the interventions on top of the other performance metrics reported and the challenges faced in this study which compromised its interpretation. For example, the decision thresholds of the algorithms could not easily be informed by the AUC as there was also a necessity for the wearable-based AI algorithm to be sensitive enough to catch (asymptomatic) infections on top of what would normally be reported through symptom evaluation. In addition, given the fundamental differences between the control and experimental condition, the AUC had little added value in the comparison or evaluation of these algorithms. Nevertheless we agree that sharing the AUC for transparency and reproducibility reasons would be an improvement of the manuscript. However, while it is indeed the case that sharing the AUC would not reveal trade secrets, the company that owns the algorithm code (Ava AG) has been acquired by another company and all individuals who have contributed to the current study have subsequently left that company. This has prevented us from even discussing the possibility of sharing the model probability outputs, which were not shared during the study as these were considered proprietary. We thus unfortunately do not have access to the probability outputs needed to calculate the AUC. The authors have investigated if there were any ways to still report the AUC, but are unable to do so at this moment, unfortunately. We are aware that this is a limitation to our manuscript, as it leaves a gap in the algorithm evaluation. We have made some adjustments to the manuscript to address this, which are explained below.*

*Firstly, the reviewer noted that the methods still listed AUC as a metric of interest, which was incorrect. We have removed this metric from the list in line 325 of the manuscript.*

*Additionally, we have further addressed the limitation of not reporting the AUC in the discussion of our manuscript by acknowledging that we cannot fully compare the performance of this study’s algorithm to that of previous literature (line 442). However, given the extremely low specificity, it can still be concluded that the study algorithm does not perform well enough to be used in practice, even without calculating the AUC.*
